# Baseline gut microbiota profiles affect treatment response in patients with depression

**DOI:** 10.3389/fmicb.2024.1429116

**Published:** 2024-07-03

**Authors:** Yingjing Xie, Hanwen Zhu, Yanling Yuan, Xuan Guan, Qinglian Xie, Zaiquan Dong

**Affiliations:** ^1^West China Hospital, Sichuan University, Chengdu, China; ^2^Department of Pharmacy, West China Hospital, Sichuan University, Chengdu, China; ^3^Chengdu Medical College, Chengdu, China; ^4^Department of Outpatient, West China Hospital, Sichuan University, Chengdu, China; ^5^Department of Psychiatry and National Clinical Research Center for Geriatrics, West China Hospital, Sichuan University, Chengdu, China; ^6^Mental Health Center, West China Hospital, Sichuan University, Chengdu, China

**Keywords:** major depressive disorder, gut microbiota, gut-brain axis, psychobiotic, treatment response

## Abstract

The role of the gut microbiota in the pathophysiology of depression has been explored in numerous studies, which have confirmed that the baseline gut microbial profiles of patients with depression differ from those of healthy individuals. The gut microbiome affects metabolic activity in the immune and central nervous systems and regulates intestinal ecology through the neuroendocrine system. Additionally, baseline changes in the gut microbiota differed among patients with depression who demonstrated varying treatment response. Currently, probiotics are an emerging treatment for depression; however, the efficacy of modulating the gut microbiota in the treatment of depression remains uncertain. Additionally, the mechanisms by which changes in the gut microbiota affect treatment response in patients with depression remain unclear. In this review, we aimed to summarize the differences in the baseline gut microbiota between the remission and non-remission groups after antidepressant therapy. Additionally, we summarized the possible mechanisms that may contribute to antidepressant resistance through the effects of the gut microbiome on the immune and nervous systems, various enzymes, bioaccumulation, and blood–brain barrier, and provide a basis for treating depression by targeting the gut microbiota.

## 1 Introduction

Depression is a psychiatric disorder characterized by various symptoms, including depressed mood, anhedonia, appetite changes, sleep disturbances, psychomotor retardation and/or agitation, fatigue, feelings of guilt, poor concentration, suicidal ideation, and cognitive impairment (GBD 2017 Disease and Injury Incidence and Prevalence Collaborators, 2018; Burrows et al., 2020). The etiology of depression involves abnormal neuroendocrine (Farzi et al., 2018), neuroimmune (Birmann et al., 2021), metabolic (Lukić et al., 2022), and neurotransmitter (Cammisuli et al., 2022) functioning. Epidemiological studies have estimated the global prevalence of depression to be 4.4% (Ferrari et al., 2013), affecting approximately 350 million people worldwide (Joca et al., 2015). According to the World Health Organization World Mental Health survey, the estimated lifetime prevalence of depression is 14.6% in high-income countries and 11.1% in low- and middle-income countries (Kessler and Bromet, 2013). In China, the prevalence of depression is 6.87%, affecting approximately 90 million people (Liang et al., 2018). Epidemiological surveys have shown that a substantial proportion of patients with major depressive disorder (MDD) fail to respond to current first-line antidepressants, imposing a substantial healthcare burden on society (Zhdanava et al., 2021). According to a meta-analysis, only 46% of individuals achieved remission by the end of treatment, even when a combination of psychotherapy and pharmacotherapy was used (de Maat et al., 2007). In addition, the adverse effects of antidepressants cause many patients to avoid these treatment options. The clinical treatment of depression includes drug therapy, cognitive behavioral therapy, physical therapy, exercise therapy, and acupuncture (Guideline Development Panel for the Treatment of Depressive Disorders, 2022; Qaseem et al., 2023), with antidepressants being the most common treatment method (Cipriani et al., 2018).

Patients with depression may not respond to treatment due to the inherent environmental and biological aspects of the disease (Minelli et al., 2022), including genetic predisposition (Pettai et al., 2016; Fabbri et al., 2018; Minelli et al., 2022), inflammatory factors (Schmidt et al., 2016; Szałach et al., 2019), thyroid autoimmunity (Dwyer et al., 2020), neurotrophic factors (Pettai et al., 2016), and dietary influences (Molendijk et al., 2018; Chakraborti et al., 2021; Marx et al., 2021; Herselman et al., 2022; Wang et al., 2022). In recent years, numerous studies have shown an association between the gut microbiota and patient response to depression treatment. Some studies have analyzed the fecal microbiota of patients undergoing treatment for depression, and found that the baseline composition of the gut microbiota differed between the two groups (Liskiewicz et al., 2021; Lee et al., 2022). Here, the findings of studies investigating the relationship between the gut microbiota and prognosis of depression are summarized to provide a basis for the treatment of depression by targeting the gut microbiota.

## 2 The gut–brain axis

The gut microbiota in adult humans includes bacteria, viruses, fungi, archaea, and protozoa (Thursby and Juge, 2017). The gut microbiota is predominantly composed of six phyla (Firmicutes, Bacteroidetes, Actinobacteria, Proteobacteria, Fusobacteria, and Verrucomicrobia), with Firmicutes and Bacteroidetes constituting the major phyla (Laterza et al., 2016; Sherwin et al., 2016). Individuals have unique gut microbiome profiles which depend on various factors, including the intestinal environment, hormonal changes, immunity, lifestyle, dietary habits, and drug use (Wang and Liu, 2020; Perler et al., 2023).

Some studies have suggested that the gut microbiota interacts with the central nervous system (CNS) through the gut–brain axis (Góralczyk-Bińkowska et al., 2022; Kim et al., 2023; Borrego-Ruiz and Borrego, 2024). Evidence supports the idea that the gut microbiota may regulate higher-level CNS functions, such as behavior and mood, through bidirectional signal transmission (Sherwin et al., 2016).

**Figure 1:**
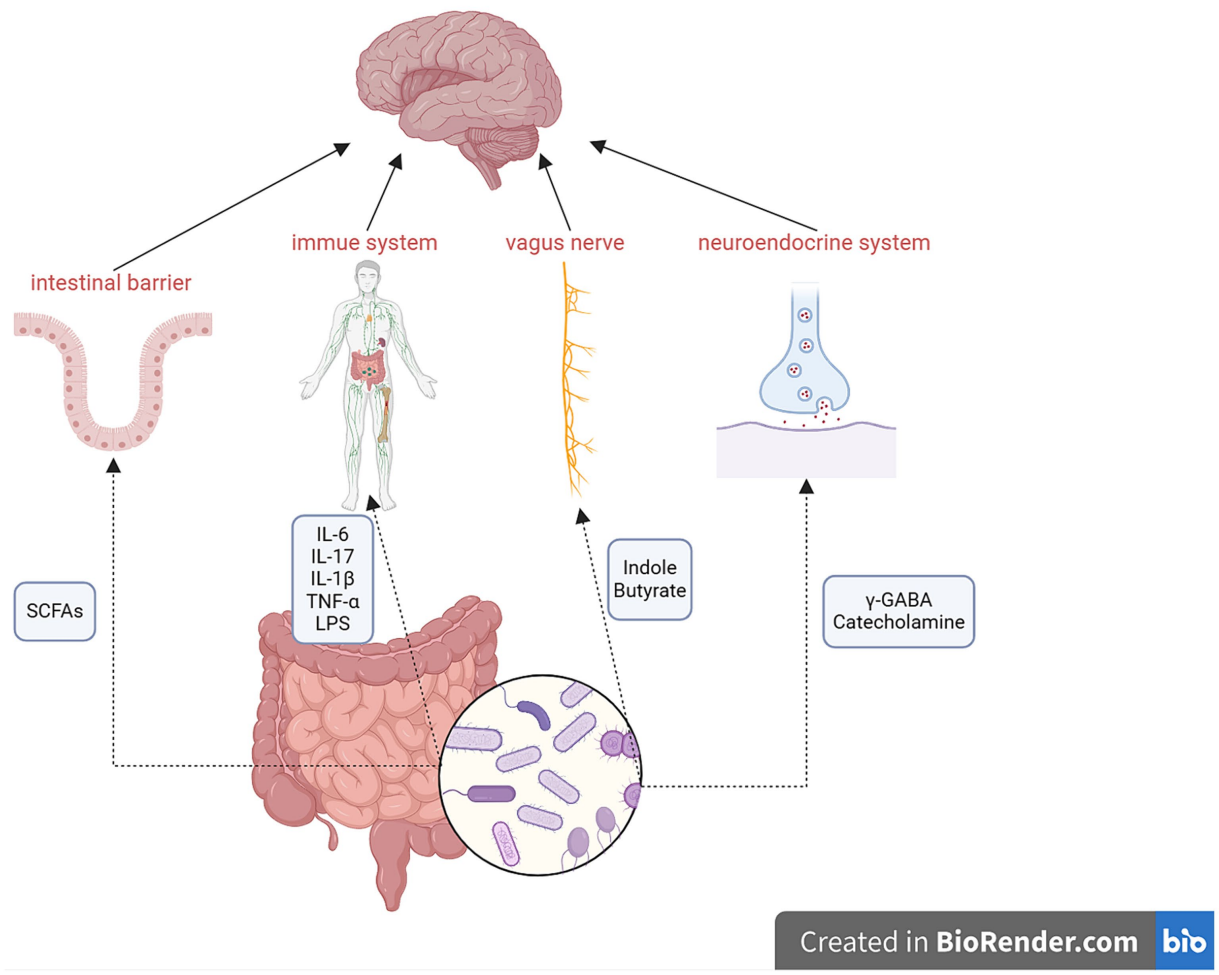
Mechanisms of the gut–brain axis. Metabolites of the gut microbiota can affect the intestinal barrier, immune system, vagus nerve, and neuroendocrine system to regulate the function of the brain via the gut–brain axis. Conversely, the brain can also influence the gut microbiota. SCFAs, short-chain fatty acids; γ-GABA, γ-aminobutyric acid.

Numerous studies have observed two-way communication between the microbiota and the brain (Kunugi, 2021; Sharma et al., 2021; Trzeciak and Herbet, 2021; Ye et al., 2021; Hou et al., 2022; Dziedzic et al., 2024) through the gut–brain axis, which encompasses multiple components, including the CNS, spinal cord, autonomic nervous system, enteric nervous system, immune system, and hypothalamic–pituitary–adrenal axis (Carabotti et al., 2015). The gut microbiota can produce various molecules that act at distal sites to imitate the function of endocrine organs (Tsigos et al., 2000; Clarke et al., 2014), such as short-chain fatty acids (SCFAs), neurotransmitters (including serotonin, dopamine, norepinephrine, and γ-aminobutyric acid), cholic acids, tryptophan, L-dopa, adipokines, and hormones (Clarke et al., 2014). The mechanisms of action of the gut–brain axis are shown in Figure 1.

Genes, socioeconomic status, diet, medications, and environmental factors can influence the gut–brain axis. Lifestyle factors, especially diet, are crucial in regulating the gut–brain axis. Additionally, drugs, especially antibiotics, can directly affect the gut microbiota, which, in turn, can impact the gut–brain axis (Hou et al., 2022).

## 3 Baseline changes in gut microbiota during different responses to depression

Several studies have reported the differences in the gut microbiota profiles of patients with depression and healthy individuals (Jiang et al., 2015; Trzeciak and Herbet, 2021). Recent studies have shown that, after treatment, the gut microbiota differs between patients who achieve clinical remission and those who do not (Liskiewicz et al., 2021; Ye et al., 2021; Lee et al., 2022). Individuals who achieved remission had a higher baseline abundance of their gut microbiota compared to non-remitters (Wang et al., 2023). The baseline gut microbiota was defined as the first gut microbiota measurement after enrollment and before initiation of antidepressant treatment.

### 3.1 Methods for evaluating response to depression treatment

The treatment response to depression is primarily evaluated using clinical scales, with treatment effectiveness evaluated by changes in the scores after treatment. Therefore, reliable and valid instruments are necessary for diagnosing and treating depression.

Over the past few decades, various instruments have been developed to assess the severity of depressive symptoms, including the Hamilton Depression Rating Scale (HAMD) (Hamilton, 1960), Montgomery–Asberg Depression Rating Scale (MADRS) (Montgomery and Asberg, 1979), and Beck Depression Inventory (Beck et al., 1961). The first two are observer rating instruments, while the latter is a self-rating measure.

The HAMD is the most commonly used scale for clinically assessing depression (Hamilton, 1960). The original version included 17 questions related to depression; however, the number of items gradually increased to 24 as the understanding of the disease improved; currently, there are three versions of the HAMD, with 17 (HAMD-17), 21 (HAMD-21), and 24 (HAMD-24) questions. The HAMD-24 comprises 24 questions related to depression, with most items scored on a five-point scale ranging from zero to four points. A total score of >35 indicates MDD. Remission from depression is defined as HAMD-24 score ≤ 8 at the end-point (Schramm et al., 2020). The MADRS consists of 10 questions, each scored from zero to six (Montgomery and Asberg, 1979). A total score of ≥30 indicates MDD, and a total score of ≥12 suggests remission (Bharwani et al., 2020).

Treatment response can also be defined as a reduction of 50% or more from the baseline score on standardized scales, such as HAMD and MADRS, after treatment for depression (Keller, 2003). Some studies have evaluated treatment response by observing animal behavior before and after treatment as well as through specific experiments, such as open-field, tail suspension, and forced swimming tests (Ding et al., 2021; Song et al., 2022).

### 3.2 Comparison of baseline changes in the gut microbiota between patients with varying response to treatment

#### 3.2.1 Changes in the level of alpha and beta diversity

Alpha diversity, which considers richness and the relative abundances of species, can be used to compare sample groups. Specifically, low alpha diversity is a sign of dysbiosis. Gut microbiota can be divided into different operational taxonomic units (OTUs) using alpha diversity (Knight et al., 2018). Beta diversity calculates the mean value of species divergence between the focal and neighboring samples. It captures the dissimilarity between a pair of samples or communities, thereby generating a distance matrix based on the presence or absence of species abundance data (Nguyen et al., 2021). Some studies have revealed no significant community-level alpha or beta diversity changes between remission and non-remission groups (Sanada et al., 2020; Liskiewicz et al., 2021; Lee et al., 2022). However, other clinical studies have found a negative association between microbial alpha diversity and depression severity (Madan et al., 2020; Shen et al., 2021).

Some studies have revealed that remitters exhibit greater baseline diversity than non-remitters (Jiang et al., 2015; Bharwani et al., 2020; Duan et al., 2021). In one study, patients with MDD were divided into the remitter and non-remitter groups based on their MADRS scores after treatment with escitalopram. OTU levels were examined after 3 and 6 months of treatment, and it was found that OTUs were not altered in non-remitters. Overall, 35 OTUs differed between remitters and non-remitters at 3 months; however, 16 OTUs were no longer different after 6 months. At 6 months, 42 OTUs differed, of which 23 were unique, and 19 were the remaining OTUs at 3 months. The results indicated that antidepressants could affect the gut microbiota of patients with MDD at the OTU level (Bharwani et al., 2020). Another study revealed that 125 and 87 OTUs were uniquely present in non-remission and remission groups, respectively (Duan et al., 2021).

#### 3.2.2 Changes in the phylum Firmicutes

Another study revealed that 125 and 87 OTUs were uniquely present in non-remission and remission groups, respectively (Duan et al., 2021). Clostridiales was strongly negatively correlated with the severity of depression (Liskiewicz et al., 2021; Xiong et al., 2022; Wang et al., 2023). An increase in the abundance of the order Clostridiales was found in the remission group after 6 months of escitalopram treatment (Bharwani et al., 2020). The abundance of *Faecalibacterium* negatively correlated with the severity of depressive symptoms (Jiang et al., 2015), and lower levels of *Faecalibacterium* were associated with MDD development (Kelly et al., 2016; Zheng et al., 2016; Cheung et al., 2019). Animal studies showed that the ingestion of *Faecalibacterium prausnitzii* improved anxiety- and depression-like behaviors in chronic unpredictable mild stress (CUMS) mice (Hao et al., 2019). In one study, the enrichment of baseline *Faecalibacterium* levels in levomilnacipran-treated patients with MDD aged >60 years was associated with remission outcomes (Lee et al., 2022). The abundance of *Faecalibacterium* was lower in MDD patients than in healthy controls, but was restored after sertraline treatment (Zhou et al., 2023). Higher abundances of *Faecalibacterium* were also associated with higher quality of life indicators (Valles-Colomer et al., 2019).

In addition, enrichment of *Agathobacter* (Lee et al., 2022), *Coprococcus* (Valles-Colomer et al., 2019; Gao et al., 2023), *Roseburia* (Zhou et al., 2023), and *Eubacterium* (Xiong et al., 2022; Wang et al., 2023) was associated with the treatment outcome of remission. The increased relative abundances of the family Christensenellaceae (Dong et al., 2022) and decreased relative abundances of the families Ruminococcaceae, Lactobacillaceae, and Peptostreptococcaceae and the genera *Coprococcus* (Sanada et al., 2020), *Oscillibacter* (Xiong et al., 2022), *Tyzzerella*, and *Butyricicoccus* (Shen et al., 2023) were related to non-remission (Fontana et al., 2020; Duan et al., 2021).

Firmicutes are important butyrate-producing bacteria (Kim et al., 2020). Butyrate was reported to reduce depression-like behaviors, produce antidepressant effects, and relieve symptoms of depression (Zhou et al., 2018).

#### 3.2.3 Changes in the phylum Bacteroidetes

The severity and treatment response of depression are strongly correlated with Paraprevotella abundance (Lin et al., 2017; Liskiewicz et al., 2021; Hu et al., 2023). An experiment using CUMS mice showed an increase in the relative abundance of *Prevotellaceae_UCG-003* in the remission group but not in the non-remission group (Duan et al., 2021). Clinical studies showed that the abundance of Bacteroidota, especially *Odoribacter*, was higher in responders (Matsuzaki et al., 2024). By contrast, another study found that the baseline abundance of Bacteroidetes gradually decreased after treatment with vortioxetine hydrobromide, consistent with the remission of depressive symptoms (Ye et al., 2021).

*Parabacteroides* protect the nervous system by altering the levels of neurotransmitters, including glutamate and γ-aminobutyric acid, in the hippocampus(Liskiewicz et al., 2021). *Odoribacter* might generate butyrate by accelerating L-lysine degradation, which can promote the barrier function of gut epithelium (Matsuzaki et al., 2024; Vasileva et al., 2024). The level of L-lysine was proved to be lower in responders after antidepressive treatment(Matsuzaki et al., 2024).

#### 3.2.4 Changes in the phylum Actinobacteria

The relative abundances of Actinobacteria and Eggerthellaceae are higher in non-responders than in responders (Fontana et al., 2020; Alexander et al., 2022; Dong et al., 2022). A significant increase in the proportion of *Bifidobacterium* was found after treatment with vortioxetine hydrobromide (Ye et al., 2021; Gao et al., 2023). In another study, the abundance of *Enterorhabdus* was higher in CUMS mice than in the control group. However, this increase was reversed after venlafaxine treatment (Shen et al., 2023).

#### 3.2.5 Changes in the phylum Proteobacteria

Studies demonstrated that with the remission of depressive symptoms, the abundance of Proteobacteria significantly decreases after treatment with vortioxetine hydrobromide (Ye et al., 2021). Responders demonstrated reduced Proteobacteria abundance when compared with non-responders (Fontana et al., 2020). Another study showed that puerarin relieved CUMS-induced depression-like behavior in rats and reduced the abundance of *Desulfovibrio* (Song et al., 2022). Changes in the gut microbiota of patient with depression with varying responses to treatment are shown in Table 1.

**Table 1:** Summary of the articles on changes in the baseline of gut microbiota associated with the response to treatment.

Sample size	Assessment	Treatment	Changes in the gut microbiota	Reference
LVM (*N* = 17) Placebo (*N* = 12)	HAMD Remission was defined as having a HAMD score ≤ 6	Randomized to receive either levomilnacipran or placebo for 12 weeks	No community-level alpha or beta diversity differences between groups. *Flavonifractor* abundance increased while *Roseburia* abundance decreased in the remission group.	Lee et al. (2022)
*N* = 15	MADRS Remission was defined as MADRS ≤12	Received citalopram or escitalopram for 6 months	*Clostridiales* abundance elevated in remitters.	Bharwani et al. (2020)
*N* = 16	HAMD-24 Early improvement: 20% reduction in HAMD-24 scored at 2 weeks; Response: 50% reduction in HAMD-24 score at 6 weeks; Remission: HAMD-24 score ≤ 8 at 6 weeks	Treated with escitalopram under standardized conditions	Treatment response was positively correlated with Paraprevotella and strongly negatively correlated with *Clostridiales*, *Clostridia*, *Firmicutes*, and RF32.	Liskiewicz et al. (2021)
Patients (*N* = 26) HCs (*N* = 28)	HAMD-17 MDD group: HAMD-17 score ≥ 24	Received vortioxetine hydrobromide for 4 and 8 weeks	Bacteroidetes and Proteobacteria abundances decreased while Firmicutes abundance increased, with the alleviation of depressive symptoms.	Ye et al. (2021)
*N* = 1,054	RAND-36 health-related quality of life (QoL) survey	–	*Faecalibacterium* and *Coprococcus* were associated with a higher QoL.	Valles-Colomer et al. (2019)
CUMS mice	–	Received escitalopram for 4 weeks	Escitalopram can increase the alpha diversity of the gut microbiota. Prevotellaceae_UCG-003 abundance increased in the remission group while that of Ruminococcaceae and Lactobacillaceae decreased relative to that in the non-remission group.	Duan et al. (2021)
P (*N* = 63) HC (*N* = 30)	HAMD-24 The responder group was defined as a 50% reduction of the score	All patients received one type of SSRI or venlafaxine for 8 weeks	Baseline abundances of Actinobacteria, Christensenellaceae, Eggerthellaceae, *Adlercreutzia*, and Christensenellaceae R7 groups were significantly lower in the remission group.	Dong et al. (2022)
CUMS mice Control: 10 CUMS: 10 Venlafaxine: 10	–	–	CUMS mice had decreased *Blautia*, *Oscillibacter*, *Tyzzerella*, and *Butyricicoccus* abundances and increased *Enterorhabdus* abundance, and all changes could be reversed by venlafaxine treatment.	Shen et al. (2023)
CUMS model	Open-field test; sucrose preference test	XYS high-dose group, XYS medium-dose group, XYS low-dose group, model group, fluoxetine group	Improvement of depression-like behavior in CUMS was related to increased levels of *Roseburia* sp. and *Eubacterium* sp.	Xiong et al. (2022)
CUMS model	Open-field test; sucrose preference test; forced swimming test	(*N* = 6 per group) Control group; CUMS group; fluoxetine group; puerarin low-dose group; puerarin high-dose group.	Puerarin reduced CUMS-induced depressive-like behavior and the abundances of *Desulfovibrio*, Verrucomicrobiae, and Verrucomicrobia.	Song et al. (2022)
29 patients with schizophrenia	HAMD 25% or more reduction of HAMD score at 4 weeks from baseline was considered to indicate a responder	*Bifidobacterium* each day for the first 4 weeks	Responders had higher lipid and energy metabolism at baseline than did non-responders.	Yamamura et al. (2021)
Male C57BL/6 mice with chronic restraint stress (CRS)	Open-field test; tail suspension test; forced swimming test	(6 per group) Control group; CRS group; CRS + AKK; AKK group; CRS + *Lactobacillus* L; CRS + *Lactobacillus* H	*Akkermansia muciniphila* reduced depressive-like behaviors of CRS mice and regulated the structure and metabolism of the gut microbiota under chronic stress.	Ding et al. (2021)
SSRI with the probiotic *Lactobacillus plantarum* 299v (*n* = 40) SSRI with placebo (*n* = 39)	HAMD-17	Participants received either an SSRI with the probiotic *Lactobacillus plantarum* 299v for 8 weeks or an SSRI with the placebo of the probiotic for the same period.	*Lactobacillus plantarum* 299v improved cognitive performance and decreased kynurenine levels in patients with MDD. No significant changes were observed in the treatment effect in either probiotic or placebo groups.	Rudzki et al. (2019)
Patients (*N* = 34) HCs (*N* = 20)	Assessment of the response was based on a retrospective assessment of longitudinal clinical course and evaluation of treatment response patterns	–	Proteobacteria, Tenericutes, and Peptostreptococcaceae levels increased in non-responders, whereas Actinobacteria levels increased in responders.	Fontana et al. (2020)
Patients (*N* = 110) HCs (*N* = 166)	HAMD-17 Remission was defined as having a HAMD score of seven or lower	Patients were treated with escitalopram for 12 weeks	Clostridiaceae decreased in the remission group when compared with that in the non-remission group. *Eubacterium hallii* increased in the remission group and decreased in the non-remission group.	Wang et al. (2023)
*N* = 34	HAMD; HAMA Responders were defined as HAMA or HAMD <8 at time point 3; remitters were defined as HAMA or HAMD <8 at time point 1	Patients were treated with antidepressants and antipsychotics	*N*-ε-acetyllysine, cysteate, glycyl-L-leucine, taurine, guanosine, L-lysine, and malonate levels were lower in responders. Bacteroidota abundances, especially that of *Odoribacter* were higher in responders.	Matsuzaki et al. (2024)
*N* = 104	HAMD-17 Responders were defined as demonstrating a minimum 50% reduction in their HAMD-17 scores after treatment	Patients received paroxetine treatment for 8 weeks	Baseline IL-6 levels were negatively correlated the reductions in HAMD-17 scores.	Dong et al. (2021)
*N* = 62	HAMD-17 Responders were defined as exhibiting a ≥ 50% reduction in their HAMD-17 scores after antidepressant treatment	Patients were treated with fluoxetine treatment for 8 weeks	Relative abundances of *Blautia*, *Coprococcus*, and *Bifidobacterium* were positively correlated with the efficacy of SSRI antidepressants.	Gao et al. (2023)
CUMS model	Sucrose preference test; forced swimming test	Negative control group; CUMS model group; paroxetine group (paroxetine 5 mg/kg)	TGF-β, IL-22, IL-10, and IL-17 levels were significantly lower in rats who responded to paroxetine than those in non-responders. Propionate, lactate, isoleucine, and citrulline levels significantly increased in responders to paroxetine therapy.	Liu et al. (2024)

MDD, major depressive disorder; HAMD, Hamilton Depression Rating Scale; HAMA, Hamilton Anxiety Rating Scale; MADRS, Montgomery–Asberg Depression Rating Scale; CUMS, chronic unpredictable mild stress; HC, healthy control; P, patients; SSRI, selective serotonin reuptake inhibitor; XYS, Xiaoyao San.

### 3.3 Possible mechanisms of how the gut microbiota affect treatment response in depression

Although the interactions between drugs and gut microbiota exist, the underlying mechanisms remain unclear.

#### 3.3.1 Gut microbiota affects drug pharmacokinetics

Hepatic metabolites of drugs are secreted into the gut for further metabolism. Bacterial metabolites of a drug can, in turn, be absorbed and transported to the liver. During this process, drugs interact with the gut microbiota. However, drugs can alter the intestinal microenvironment and/or directly affect the growth, composition, and function of bacteria (Weersma et al., 2020). Some antidepressant drugs, such as sertraline, fluoxetine, and escitalopram, exert antibacterial effects, disrupting the integrity and stability of the gut microbiota (Macedo et al., 2017; Karine de Sousa et al., 2018). Moreover, changes in the gut microbiota can affect drug metabolism and influence the efficacy of drugs.

A systematic analysis tested the ability of 76 gut microbial strains to metabolize 271 drugs administered orally and found that 176 drugs (66%) were metabolized by at least one microbial strain (Zimmermann et al., 2019b). The gut microbiota can alter the structure, bioavailability, biological activity, and toxicity of drugs via enzymes, directly affecting individual responses to a particular drug (Maini Rekdal et al., 2019; Zimmermann et al., 2019a,b; Weersma et al., 2020). For example, the gut microbiota can transform the chemical structure of drugs and modulate xenobiotic metabolism, including drug metabolic pathways (Wilson and Nicholson, 2017). The gut microbiota can enhance the activity of indoleamine 2,3-dioxygenase 1, a rate-limiting enzyme which transforms tryptophan into kynurenine and its derivatives, thereby affecting the bioavailability of antidepressants (Agus et al., 2018; Wu et al., 2022). Changes in the gut microbiota can also impact intestinal permeability and the function of the intestinal barrier, which affects drug absorption (Kelly et al., 2015). Firmicutes break down carbohydrates into SCFAs (Stilling et al., 2016; Deleu et al., 2021), which have been shown to enhance the integrity of the blood–brain and intestinal barriers (Unger et al., 2016). Reduced levels of Firmicutes results in decreased SCFA production (Huang et al., 2018). Additionally, gut microbiota possess tryptophanase, which can produce indole to regulate intestinal barrier permeability (Trzeciak and Herbet, 2021). Significant increases in lactate levels were observed in CUMS rats that responded to treatment (Liu et al., 2024). Supplementation with either *Bifidobacterium* or *Lactobacillus* can enhance the integrity of the intestinal barrier and alleviate the symptoms of stress-induced intestinal leakage (Couto et al., 2020; Lenoir et al., 2020).

#### 3.3.2 Gut microbiota influences immune regulation

Evidence suggests that systemic inflammation, mediated by intestinal dysbiosis, could play a crucial role in the development of therapy resistance in patients with depression (Vitetta et al., 2014). TGF-β, IL-22, IL-10, and IL-17 levels were significantly higher in CUMS rats who responded to paroxetine therapy than those in non-responders (Liu et al., 2024). Baseline IL-6 levels were negatively correlated with reduced HAMD-17 scores in clinical studies (Dong et al., 2021). *Faecalibacterium prausnitzii* produces butyrate in the human colon, and appropriate levels of butyrate production can improve mucin secretion, prevent intestinal leakage, and suppress inflammation (Furusawa et al., 2013; Samara et al., 2022). It can also regulate intestinal epithelial cells to decrease pro-inflammatory cytokine levels and increase anti-inflammatory factor levels (Couto et al., 2020; Lenoir et al., 2020). Increased *F. prausnitzii* levels can lead to increased SCFA production and higher levels of inflammatory factors, such as IL-10, in the plasma (Hao et al., 2019). Microbiota metabolites can also modulate the proportions of T helper 17 and regulatory T cells to promote resilience to stress-induced depressive-like behaviors (Westfall et al., 2021). High levels of Prevotellaceae UCG-003 may regulate intestinal inflammation by producing succinate, which, in turn, activates dendritic cells (Koh et al., 2016). Bifidobacteria increase butyrate levels by altering the relative abundances of other microbiota involved in lipid metabolism, therefore possessing anti-inflammatory properties (Sugahara et al., 2015). Moreover, Bifidobacteria regulate the levels of pro-inflammatory cytokines and anti-inflammatory factors (Couto et al., 2020; Lenoir et al., 2020; Samara et al., 2022). Furthermore, members of the genus *Eggerthella*, including *Eggarthella lenta*, induce intestinal inflammation by activating Th17 cells (Alexander et al., 2022).

#### 3.3.3 Gut microbiota affects the nervous system

The gut microbiota is essential for nervous system communication via the vagus nerve. The vagus nerve plays an important role in behavioral abnormalities in antibiotic-treated mice after ingesting *Lactobacillus* (Wang et al., 2020). In animal models, dysbiosis impairs vagus signaling, affects brain structure, regulates brain-derived neurotrophic factors, and reduces hippocampal protein synthesis (Fond et al., 2015; Dehghani et al., 2022). The relative abundance of Proteobacteria is correlated with stress-induced behavioral changes (Wong et al., 2016; Werbner et al., 2019). Members of the genus Bifidobacteria play crucial roles in maintaining the balance of the gastrointestinal tract by reducing oxidative stress (Kant et al., 2015). Additionally, *Oscillibacter* is beneficial in protecting brain modulatory functions, ultimately leading to increased amygdala and hippocampal volume (Lee et al., 2022). *Parabacteroides* can alter neurotransmitter levels in the brain, including those of glutamate and aminobutyric acid (Olson et al., 2018). Furthermore, *Bifidobacterium* and *Lactobacillus* stimulate γ-aminobutyric acid production by metabolizing indigestible fibers (Couto et al., 2020; Lenoir et al., 2020).

#### 3.3.4 Other effects of the gut microbiota

**Figure 2:**
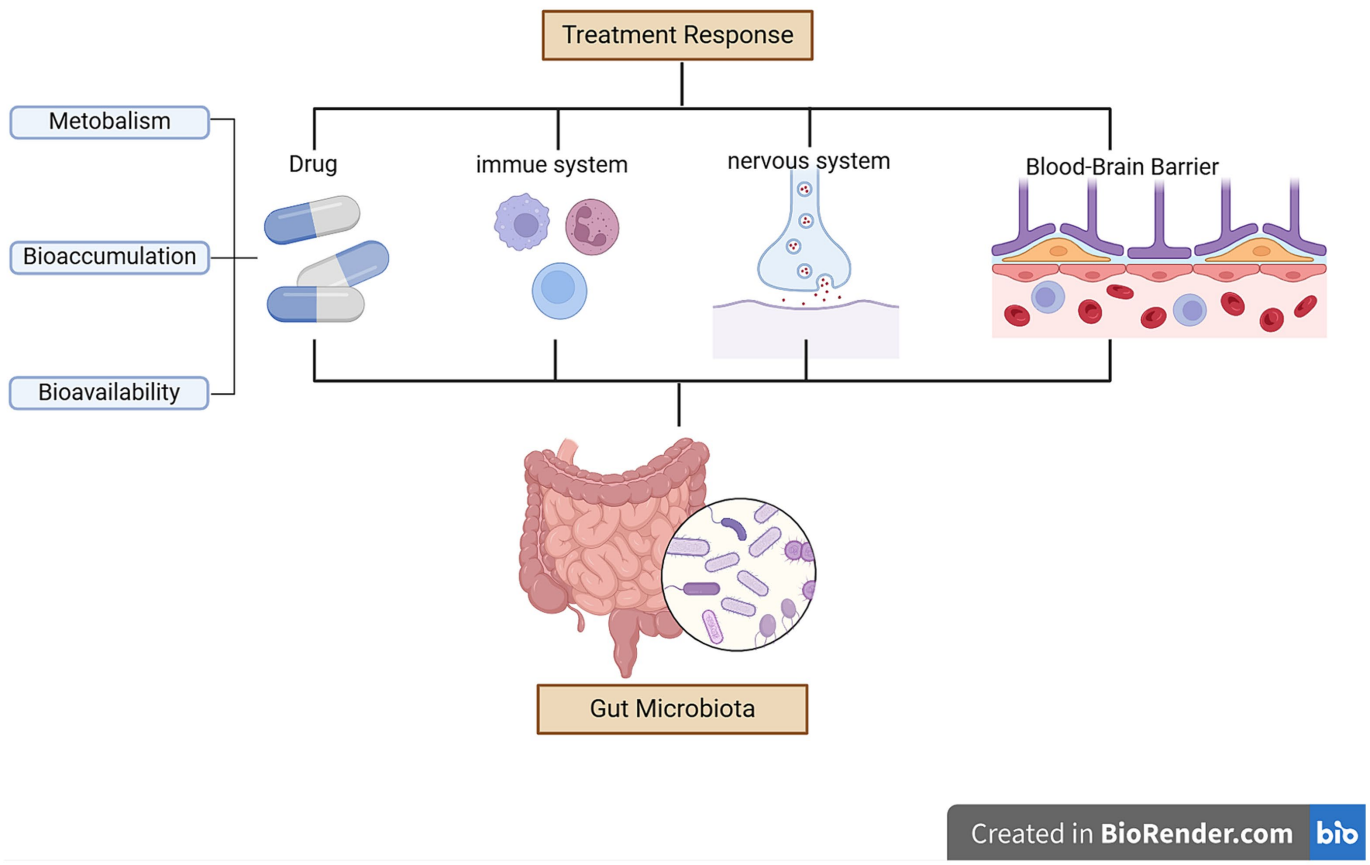
Mechanisms underlying how the gut microbiota can affect treatment response in depression.

The gut microbiota may affect the therapeutic outcomes of antidepressants by altering the permeability of the blood–brain barrier permeability during treatment (Xu et al., 2023). Higher abundances of Firmicutes were associated with higher levels of SCFAs (Stilling et al., 2016; Deleu et al., 2021). SCFAs can enhance the integrity of the blood–brain barrier (Unger et al., 2016). The gut microbiota can modulate the availability of antidepressants through bioaccumulation (Doestzada et al., 2018; Klünemann et al., 2021), resulting in direct reductions in drug availability and changes in the secretion of metabolites (Wu et al., 2017; Klünemann et al., 2021). *Streptococcus salivarius*, *Bacteroides uniformis*, *Bacteroides thetaiotaomicron*, and *Escherichia coli* increase duloxetine bioaccumulation, thereby decreasing its bioavailability (Klünemann et al., 2021). Figure 2 summarizes the possible mechanisms by which the gut microbiota affects treatment response in patients with depression.

### 3.4 Current progress in microbiota-targeting therapies

Some studies using adjuvant microbial supplements for the treatment of depression have achieved preliminary success (Rieder et al., 2017); Current research findings indicate that probiotic therapy may have moderate efficacy in alleviating depressive symptoms (Nikolova et al., 2021; Tian et al., 2022). Most studies have utilized the genera *Lactobacilli* and *Bifidobacteria* as probiotics for treating depression. However, the effectiveness of probiotic monotherapy is limited (Nikolova et al., 2021). Since most antidepressants exhibit antimicrobial activity (Nikolova et al., 2021), probiotics can promote therapeutic benefits by restoring the balance of the gut microbiota and minimizing gastrointestinal discomfort. However, the complexity and variability of the gut microbiota, as well as its susceptibility to various influencing factors, lead to heterogenous trial results (Tian et al., 2022).

Current preclinical research findings indicate that fecal microbiota transplantation has significant potential in the treatment of MDD, but there are still limited number of studies on humans, and several issues remain, including resistance to microbiota colonization, potential pathogen transmission, and ethical considerations related to donor-recipient matching (Meng et al., 2024). Further studies on the treatment of MDD using gut microbiota are needed.

## 4 Conclusion

Multiple studies have confirmed that differences exist in the gut microbiota of patients with MDD and healthy subjects; however, they have not conclusively shown that these differences are correlated with disease severity. This review summarized the existing studies that compared the baseline gut microbiota between remission and non-remission groups and found that changes in the abundances of specific microbiota are associated with treatment response in MDD.

However, most of these studies have certain limitations. Most notably, the sample sizes are often too small to confirm significant differences between the two groups. Some trials have not included healthy controls, and some have lost a high proportion of participants to follow-up, leading to greater selection bias. Recent studies suggest that baseline changes in specific gut microbiota are related to MDD remission; however, the provided evidence is insufficient to confirm that these specific organisms can be used as predictors for the treatment response of depression. In addition, the detection of the gut microbiota is greatly affected by individual differences and environmental factors, which warrants further research.

This review provides supporting evidence for microbiota therapy and indicates novel research directions for using the gut microbiota as a target in prognosticating and treating depression. Continued research is crucial for understanding the relationship between the gut microbiota and depression, which can offer new prospects in treating this complex condition. Therapies targeting the gut microbiota are expected to be widely utilized as a treatment option for depression in the future.

## Author contributions

YX: Writing – original draft, Writing – review & editing. HZ: Methodology, Writing – review & editing, Conceptualization. YY: Methodology, Writing – review & editing, Supervision. XG: Writing – review & editing, Methodology, Validation. QX: Funding acquisition, Writing – review & editing. ZD: Conceptualization, Funding acquisition, Project administration, Writing – review & editing.
